# Reactive Oxygen Species in Regulating Lymphangiogenesis and Lymphatic Function

**DOI:** 10.3390/cells11111750

**Published:** 2022-05-26

**Authors:** Bhupesh Singla, Ravi Varma Aithabathula, Sonia Kiran, Shweta Kapil, Santosh Kumar, Udai P. Singh

**Affiliations:** 1 Department of Pharmaceutical Sciences, The University of Tennessee Health Science Center, Memphis, TN 38017, USA; drsmyrnabio@gmail.com (R.V.A.); skiran@uthsc.edu (S.K.); ksantosh@uthsc.edu (S.K.); usingh1@uthsc.edu (U.P.S.); 2 Division of Gastroenterology, Hepatology and Nutrition, Cincinnati Children′s Hospital Medical Center, Cincinnati, OH 45229, USA; shweta.kapil@cchmc.org

**Keywords:** lymphatic vessels, lymphangiogenesis, reactive oxygen species, superoxide anion, hydrogen peroxide, nitric oxide

## Abstract

The lymphatic system is pivotal for immunosurveillance and the maintenance of tissue homeostasis. Lymphangiogenesis, the formation of new lymphatic vessels from pre-existing vessels, has both physiological and pathological roles. Recent advances in the molecular mechanisms regulating lymphangiogenesis have opened a new area of research on reparative lymphangiogenesis for the treatment of various pathological disorders comprising neurological disorders, cardiac repair, autoimmune disease, obesity, atherosclerosis, etc. Reactive oxygen species (ROS) produced by the various cell types serve as signaling molecules in several cellular mechanisms and regulate various aspects of growth-factor-mediated responses, including lymphangiogenesis. The ROS, including superoxide anion, hydrogen peroxide, and nitric oxide, play both beneficial and detrimental roles depending upon their levels and cellular microenvironment. Low ROS levels are essential for lymphangiogenesis. On the contrary, oxidative stress due to enhanced ROS generation and/or reduced levels of antioxidants suppresses lymphangiogenesis via promoting lymphatic endothelial cell apoptosis and death. In this review article, we provide an overview of types and sources of ROS, discuss the role of ROS in governing lymphangiogenesis and lymphatic function, and summarize the role of lymphatics in various diseases.

## 1. Introduction

The lymphatic system, constituted by a network of lymphatic vessels (LVs), lymph nodes (LNs), and lymphoid organs, runs parallel to the blood vascular system. Both lymphatic and blood circulatory systems work in synchrony to maintain tissue homeostasis. The blood vascular system supplies nutrients, oxygen, and hormones to various body organs. In contrast, the lymphatic system plays an important role in transporting extravasated interstitial fluid, immune cells, inflammatory cytokines, antigens, and lipoproteins from the peripheral tissue to the draining LNs and back to the systemic venous circulation. Thus, the lymphatic system is pivotal for the maintenance of interstitial fluid homeostasis, host defense, adaptive immunity, and regulation of inflammatory responses [1,2,3]. The LVs are present throughout the human body with the exception of bone marrow and tissues, such as the epidermis, where blood vessels are also absent. These vessels are categorized in a hierarchical network of vessels, including capillaries, pre-collecting, and collecting lymphatics, based on their specific functions and morphological features [4,5]. Lymph flows through the lymphatic network in a unidirectional manner and at low pressure. The lymph flow rate is influenced by various extrinsic and intrinsic forces. Dysfunctional lymphatic vessels are responsible for several pathological conditions, including inherited and acquired lymphedema, malabsorption syndromes, autoimmune disease, atherosclerosis, neurological disorders, and immune deficiency [6,7,8].

The LVs are lined by a single layer of lymphatic endothelial cells (LECs), which possess specific markers and regulatory molecules, including Prospero homeobox 1 (Prox1), podoplanin, vascular endothelial growth factor receptor 3 (VEGFR3), and lymphatic vessel endothelial hyaluronan receptor 1 (LYVE-1) [9,10,11,12,13]. Like angiogenesis, lymphangiogenesis is the formation of new lymphatic vessels from pre-existing lymphatics. It results from a complex series of cellular events, including proliferation, sprouting, migration, and formation of vessel-like structures by LEC. Unlike developmental lymphangiogenesis, lymphangiogenesis in adults is dysregulated and less coordinated and occurs in pathological conditions such as inflammation, wound healing, and tumor growth [14,15,16]. These pathologies are often characterized by an accumulation of inflammatory cells and tissue edema, which necessitate lymphangiogenesis and LV remodeling for the removal of immune cells, cytokines, and tissue fluid [15,16]. Due to the various functions of LVs, lymphangiogenesis is regulated by multiple signaling pathways, as reviewed earlier [17,18].

Reactive oxygen species (ROS) such as superoxide anion, hydrogen peroxide, and nitric oxide are known to play both physiological and pathophysiological roles. Low levels of ROS are required to mediate LEC proliferation and migration, contributing to lymphangiogenesis. Increased generation of ROS by LEC leads to oxidative stress and inhibits LEC proliferation, migration, and tube formation via inducing apoptosis and cell death [19,20,21]. Based on these and the literature review, in this review article, we provide an overview of types and sources of ROS, discuss the role of ROS in governing lymphangiogenesis and lymphatic function in various pathological conditions, and summarize the role of LVs in various diseases.

## 2. Reactive Oxygen Species and Free Radicals

Reactive molecules and free radicals derived from molecular oxygen are called ROS. Free radicals are molecular species capable of independent existence that contain one or more unpaired electrons and include nitric oxide (NO^•^), superoxide anion (O_2_^•−^), hydroxyl radical (^•^OH), and lipid peroxyl radical (LOO^•^). Examples of non-radical derivatives of molecular oxygen are peroxynitrite (ONOO^−^), hydrogen peroxide (H_2_O_2_), hypochlorous acid (HOCl), and ozone (O_3_). ROS are synthesized as necessary intermediates in a broad range of biochemical processes and function as second messengers in physiological signaling mechanisms, contributing to the maintenance of tissue homeostasis [22]. In contrast, under pathophysiological conditions, overproduction of ROS and/or diminished antioxidant systems (also known as oxidative stress) may induce oxidative damage to DNA, protein, and lipid molecules. Oxidative-stress-induced alterations from physiological to pathophysiological signal transduction pathways and subsequent cellular damage play a critical role in the initiation, development, and progression of several diseases, including cardiovascular, inflammatory, neurologic, cancer, diabetes, and aging [19,23,24,25]. In this section, we briefly describe the chemistry and biochemistry of ROS and free radicals.

### 2.1. Nitric Oxide (NO^•^)

NO^•^ plays a role in several biological processes and has vasodilatory, anti-inflammatory, and anti-thrombotic activities. It is produced by endothelial nitric oxide synthase (eNOS), neuronal NOS (nNOS), and inducible NOS (iNOS) [22]. These isoforms catalyze the formation of L-citrulline from L-arginine, and NO^•^ is produced as a byproduct of the reaction. NOS-mediated NO^•^ production is dependent on the availability of oxygen, so in case of limited oxygen supply, the nitrate–nitrite–NO pathway acts as a backup system to maintain sufficient NO^•^ production [26]. Nitrate is first reduced by gastrointestinal and oral commensal bacteria to nitrite, which is further reduced to NO^•^ by various pathways, as mentioned in Table 1. The formation of the iron–nitrosyl complex by the interaction of NO^•^ to Fe^2+^ heme protein activates soluble guanylyl cyclase (sGC), which synthesizes second messenger cyclic guanosine monophosphate (cGMP). This sGC-cGMP signaling plays an important role in vasodilation, nerve signaling, mitochondrial biogenesis, angiogenesis, and lymphangiogenesis [27,28]. NO^•^ is also responsible for decreased production of O_2_^•−^ from complex I and III of the electron transport chain, mitochondrial cytochrome c release, and apoptosis [29,30]. Furthermore, NO^•^ is responsible for limiting calcium availability in vascular smooth muscle cells due to ATP-dependent potassium channel opening, which prevents myosin light chain 2 (MLC2) phosphorylation and inhibits vasoconstriction [31]. The equilibrium between NO^•^ and O_2_^•−^ production is regulated by the availability of tetrahydrobiopterin (BH_4_, a cofactor required for NOS activity). The outcomes of reduced availability of BH_4_ relative to its oxidized form, dihydrobiopterin (BH_2_), involve increased O_2_^•−^ release and decreased NO^•^ production [32]. This situation is called eNOS uncoupling, which can be culpable for more superoxide production by the activation of oxidase enzymes, particularly NADPH oxidases (NOXs) and xanthine oxidase (XO); and progressive reduction in NO^•^ bioavailability [33]. Moreover, the interaction between NO^•^ and O_2_^•−^ forms ONOO^−^, a strong oxidant that can oxidize BH_4_ and leads to enhanced eNOS uncoupling.

### 2.2. Superoxide Anion (O_2_^•−^)

O_2_^•−^ is the precursor of most ROS and is rapidly dismuted to H_2_O_2_ spontaneously or via the reaction catalyzed by superoxide dismutase (SOD). The rate of spontaneous dismutation of O_2_^•−^ is very low compared to enzymatic dismutation (8 × 10^4^ M^−1^ s^−1^ versus 2 × 10^9^ M^−1^ s^−1^) [34]. Enzymes involved in the biological production of O_2_^•−^ include NOX isoforms, XO, uncoupled eNOS, and lipoxygenase. Phagocytes involving myeloid cells-neutrophils, macrophages, and monocytes, kill invading pathogens via producing a large amount of O_2_^•−^, also called a respiratory burst. It is also formed due to the leakage of one electron from the mitochondrial electron transport chain to molecular oxygen [35]. It has been reported that approximately 1–3% of electrons leak to produce superoxide anions during the mitochondrial transport chain [36].

### 2.3. Hydrogen Peroxide (H_2_O_2_)

H_2_O_2,_ a highly stable and cell-permeable molecule, is generated because of O_2_^•−^ dismution catalyzed by SOD. It acts as a signaling molecule in cellular signal transduction pathways [37]. In addition, XO and Nox4 are direct sources of H_2_O_2_ [37,38]. It plays a role in various physiological activities involving cell differentiation, proliferation, and migration [22,39]. Due to its cell-permeable nature, it can act in a paracrine manner between various cell types to regulate cellular signaling.

### 2.4. Peroxynitrite (ONOO^−^)

It is formed because of the interaction of NO^•^ with O_2_^•−^ and can lead to increased NOS uncoupling in the endothelium. It is highly toxic and acts as a substrate for the formation of super active nitroso peroxo carboxylate (ONOOCO_2_^−^) or peroxynitrous acid (ONOOH) [40]. It is capable of altering the oxidative state of lipids, DNA, and tyrosine and methionine residues in proteins [41]. Peroxynitrite stimulates the nitration of tyrosine residues present in proteins, for instance, SOD, by reaction mediated by transition metals. [42]. Further, peroxynitrite in phagocytic cells acts as a cytotoxic effector molecule against invading pathogens, including bacteria and parasites [43,44,45].

### 2.5. Hydroxyl Radical (^•^OH)

The dismution of H_2_O_2_ (Fenton reaction) and peroxynitrous acid (ONOOH), which is formed from the oxidation of ONOO^−^, results in the generation of ^•^OH. Haber–Weiss reaction comprising spontaneous interaction of O_2_^•−^ and H_2_O_2_ is an alternate source of hydroxyl radical production [46]. Generally, ^•^OH does not play any role in cell signaling, but it is an important contributor to oxidative stress [47]. Its cellular levels can be altered by antioxidant enzymes and iron ligands.

### 2.6. Lipid Peroxyl Radical (LOO^•^)

Oxidation of unsaturated fatty acids present in cell membranes and lipoproteins leads to the formation of a lipid alkyl radical (L^•^), which rapidly reacts with molecular oxygen to form lipid peroxyl radical (LOO^•^). LOO^•^ radical reacts with various proteins and carbohydrates by lowering the activation energy even much more than enzyme-catalyzed reactions, leading to the production of corresponding carbonyl compounds [48]. These radicals are highly destructive to cells, and once produced, they may lead to unrelenting lipid breakdown [49].

### 2.7. Hypochlorous Acid (HOCl)

It is generated in inflammatory cells such as activated neutrophils, monocytes, and macrophages, which have myeloperoxidase (MPO) enzyme required to produce HOCl. It is synthesized by the interaction of H_2_O_2_ with chloride ions. This HOCl has a strong antimicrobial activity and can oxidize lipoproteins, lipids, and proteins [50,51]. HOCl can also be responsible for the formation of monochloramines (NH_2_Cl), ONOO^−^, ^•^OH, singlet oxygen (O_2_), and O_3_ via interacting with NH3 and other ROS [50].

### 2.8. Ozone (O_3_)

Ozone has a powerful oxidizing property and can increase the process of leukocytosis and phagocytosis. It is produced by electric discharge and irradiation of oxygen (air) with short-wavelength ultraviolet radiations. It may be synthesized in vivo by an antibody-mediated H_2_O oxidation pathway [52]. It can oxidize various biomolecules including lipids, proteins, and nucleic acids [53,54].

## 3. Role of ROS in Regulating Lymphangiogenesis and Lymphatic Function

Excessive production of ROS, for instance, H_2_O_2_, contributes to cell death. ROS-stimulated cell death occurs due to oxidative damage to cellular macromolecules involving proteins, lipids, and nucleic acids, and/or induction of cell-death-signaling pathways [55,56]. Alternatively, high ROS levels activate cell survival molecular-signaling cascades such as mitogen-activated protein kinase and PI3K/Akt [57,58]. The PI3K/Akt signaling prevents oxidative-stress-induced cell death and promotes cell survival. Furthermore, ROS have been shown to trigger cell surface growth factor receptor-mediated cell survival signaling in various cell types [59,60,61,62]. Thus, induction of growth factor receptor-mediated signaling in response to ROS protects against oxidative-stress-induced cell damage. Nitric oxide derived from eNOS in vascular endothelial cells plays an important role in stimulating angiogenesis and maintaining vascular contractility [63,64]. Like vascular endothelial cells, LECs have eNOS, and NO-derived from eNOS has been observed to be essential for lymphangiogenesis [65]. Despite numerous indications of the regulation of blood vessel formation (angiogenesis) by oxidative stress, the effects of ROS in modulating lymphangiogenesis are understudied to date.

### 3.1. Nitric Oxide

**Figure 1:**
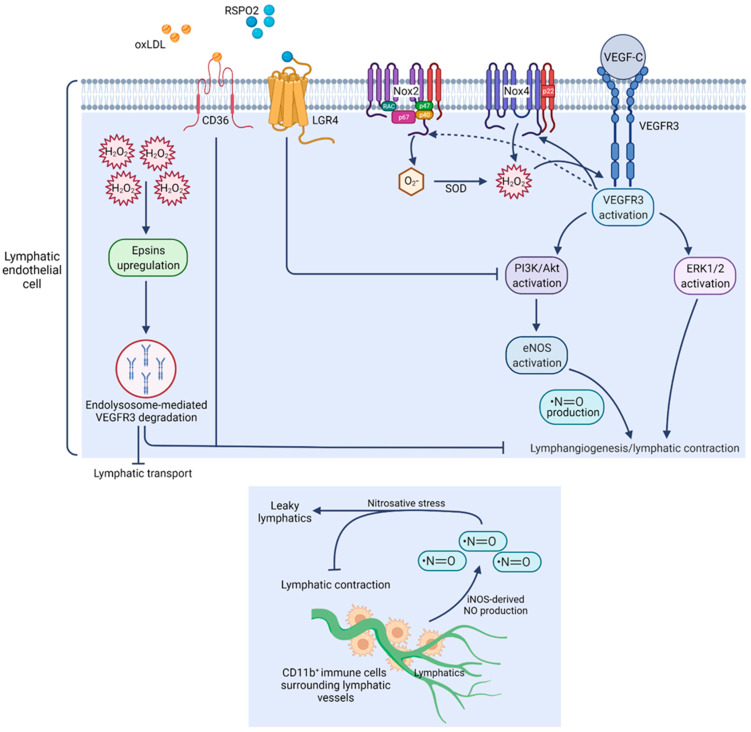
Role of ROS in regulating lymphangiogenesis and lymphatic function. Activation of VEGFR3 present in LECs by its ligand VEGF-C induces optimal Nox4-derived H_2_O_2_ production, which in turn enhances VEGFR3 autophosphorylation and stimulates downstream pro-lymphangiogenic signaling (upper panel). Oxidized LDL and RSPO2 inhibit lymphangiogenesis via suppression of Akt/eNOS pathway. Under diabetic condition, excessive H_2_O_2_ generation elevates epsin expression and promotes VEGFR3 degradation, leading to attenuated lymphangiogenesis and reduced lymphatic transport. In inflammatory condition, supra-physiological NO production by CD11b^+^ myeloid immune cells surrounding LVs contributes to nitrosative stress leading to the suppression of lymphatic contractions and inducing LV leakiness (lower panel).

Stimulation of LECs with VEGF-C, a lymphangiogenic factor, has been shown to activate eNOS, which leads to the generation of NO and a subsequent increase in LEC proliferation and lymphangiogenesis [66] (Figure 1). Under physiological conditions, various immune cells, interstitial fluid, cytokines, and antigens present in initial lymphatics are carried by collecting LVs to draining LNs. The lymph flow in collecting LVs depends on the contractility of these collecting LVs. NO-derived from LEC eNOS is important for the maintenance of lymphatic contractility [67]. In addition, NO released by LECs regulates lymphatic permeability [68,69]. Additionally, inhibition of NO production with a NOS inhibitor, L-NMMA, blocks LV regeneration. Expression/activity of NOS in tumor tissues positively correlates with lymphatic metastasis in various types of tumors [70]. Genetic deletion of eNOS and pharmacological inhibition of its activity reduces peritumoral lymphatic hyperplasia in VEGF-C-overexpressing fibrosarcoma and attenuates trafficking of tumor cells to draining LNs, suggesting the role of NO in regulating lymphatic drainage [66]. Inhibition of NO-mediated signaling using an sGC inhibitor abrogates ultraviolet B-irradiation-induced LV enlargement, edema formation, and skin inflammation in mouse models [28]. A recent study by Singla et al. reported matricellular protein R-spondin 2 (RSPO2) as an anti-lymphangiogenic protein [21]. The authors observed that RSPO2 suppresses VEGF-C-stimulated Akt and eNOS phosphorylation, leading to attenuation of NO production by LEC and subsequent impairment in lymphangiogenesis. Moreover, pharmacological NO supplementation using an NO donor prevented the inhibitory effects of RSPO2 on lymphangiogenesis [21]. All this information suggests that NO-sGC signaling is pivotal in regulating lymphangiogenesis and maintenance of LVs. Increased iNOS levels disrupt endogenous NO gradients normally regulated by eNOS and lead to supra-physiological levels of NO, which in turn results in the induction of nitrosative stress [71]. Liao et al. demonstrated that under inflammatory conditions, NO derived from iNOS-overexpressing CD11b^+^ Gr-1^+^ myeloid-derived suppresser cells present around the subcutaneous LVs suppresses lymphatic contractions [72] (Figure 1). A study by Rehal et al. reported leaky and dilated LVs surrounded by iNOS^+^ CD11b^+^ inflammatory cells in obese mice [73]. These inflammatory cells caused an increased generation of peroxynitrite in obese mice. Additionally, LECs isolated from obese mice exhibited reduced VEGFR3 and podoplanin expression. These findings suggest that enhanced iNOS-derived NO generation in obesity contributes to lymphatic injury and impaired lymphatic pumping via nitrosative stress. Other reports revealed inhibition of lymphatic contractile function with high NO levels and impaired NO signaling in diabetic condition [74,75,76]. Additionally, reduced NO production due to decreased eNOS levels in the lymphatic thoracic duct isolated from rats with metabolic syndrome has been shown to be responsible for reduced vessel contractility [77]. Morris et al. showed KLF2-PPAR-γ-signaling-dependent elevation of NOX-derived ROS production and reduction of bioavailable NO in response to shear stress as observed with chronically increased lymph flow [78]. Further, it has been shown that shear stress reduces eNOS expression and activity in LECs [79].

### 3.2. Superoxide Anion and H_2_O_2_

Increased generation of O_2_^•−^ scavenges NO and reduces NO bioavailability. Interaction of NO with O_2_^•−^ forms peroxynitrite (ONOO^−^), which can further enhance protein modification and DNA damage. Singla et al. observed Nox4 as the major NOX isoform in LECs, which mainly generates H_2_O_2_ [21]. Exposure of LECs to H_2_O_2_ induces VEGFR3 activation and downstream Akt phosphorylation [80]. VEGFR3 activation following H_2_O_2_ treatment occurs as a compensatory mechanism to promote the survival of LECs, and treatments with antioxidants including N-acetylcysteine and catalase prevent H_2_O_2_-stimulated VEGFR3 activation [80]. Considering the importance of VEGFR3 activation in the development and maintenance of the lymphatic system, these findings may be the key to understanding the pathogenesis of lymphatic-related diseases such as lymphedema. Hereditary lymphedema patients are susceptible to ROS-induced cell damage. Elevated ROS generation and augmented lipid peroxidation occurring in lymphoedematous tissue are considered to induce lymphatic endothelium damage [81,82]. Wu et al. reported that LECs isolated from diabetic mice have high oxidative stress compared with cells isolated from control mice, and LEC ROS levels inversely correlate with both in vitro and in vivo lymphangiogenesis [19]. Mechanistically, the authors found that hyperglycemia-stimulated ROS generation induces c-Src-dependent upregulation of epsins, which are responsible for the degradation of VEGFR3 in LECs. Further, treatment of LECs with a high concentration of H_2_O_2_, as observed in diabetes, causes VEGFR3 phosphorylation and degradation in a ligand-(VEGF-C) independent manner [19]. These studies demonstrate that cell surface VEGFR3 expression in LECs plays a beneficial role in protecting against oxidative-stress-induced cell damage, and loss of VEGFR3 promotes pathological conditions [19,80].

Zawieja et al. reported attenuated lymphatic pumping after exposure to hypoxanthine and xanthine oxidase, which stimulate the generation of O_2_^•−^ and H_2_O_2_. Moreover, treatment with SOD partially prevented the effects of hypoxanthine/xanthine oxidase on lymphatic contractions [83]. In another study, Zawieja and Davis investigated the effects of H_2_O_2_ challenge on the active pumping activity of mesenteric collecting lymphatics and observed significantly declined contraction frequency and lymph flow in H_2_O_2_-exposed lymphatics [84]. Further, a previous study has shown increased permeability of microvasculature in response to xanthine treatment, and treatment with O_2_^•−^ and ^•^OH scavengers reduced microvascular permeability [85]. In aging, there is a progressive decrease in NO levels and a concurrent increase in the production of free radicals. Aging-associated oxidative stress may be related to reduced levels of antioxidant enzymes such as SOD and/or increased ROS production [86,87]. A previous report suggested that aging remarkably reduces mesenteric LV contractility, which may limit the ability of these vessels to clear interstitial fluids and inflammatory cells from the site of inflammation in elderly individuals [88]. A subsequent study revealed elevated ROS production and reduced antioxidant enzyme levels in LECs isolated from aged rats, which suggests that aging-associated oxidative stress may contribute to lymphatic pump dysfunction in the elderly [89]. Thus, both O_2_^•−^ and H_2_O_2_ play inhibitory roles in governing lymphatic pumping. A recently published study showed that oxidized low-density lipoprotein (oxLDL) inhibits lymphangiogenesis, blocks cell cycle progression, reduces expression of Akt and eNOS, increases p27 (an inhibitor of the cell cycle) expression, and induces intracellular ROS generation in LECs [20]. Additionally, CD36 knockdown in LECs prevents oxLDL-induced suppression of lymphangiogenesis. However, it is not known whether scavenging ROS can prevent oxLDL′s effects on lymphangiogenesis.

ROS production is high in the colon of IBD patients, and its levels correlate with the incidence of colitis [90,91]. However, in the case of IBD, elevated inflammation-induced LV density and defective lymphatic drainage have been observed compared with controls [92,93]. Further, Nox1 is highly expressed in colon cancer and supports tumor growth. Stalin et al. have recently demonstrated that inhibition of Nox1-mediated signaling via genetic deletion and pharmacological approach (GKT771) reduces lymphangiogenesis, suppresses recruitment of proinflammatory macrophages, and retards tumor growth [94], suggesting that ROS contribute to colon lymphangiogenesis in the cancer setting. Considering the findings of Wang et al., it is possible that ROS contribute to lymphangiogenesis via VEGFR3 activation in tumor tissue [80]. Recently, it has been demonstrated that Nox4 promotes both VEGF-C-dependent and -independent lymphangiogenesis [95]. Additionally, there is evidence of engagement of Nox2-derived ROS in regulating lymphangiogenesis [96]. LPA-stimulated ROS production, which is mediated by phospholipase C and protein kinase C, regulates LPA 1/3-dependent VEGF-C expression in prostate cancer cells [97]. LPA stimulates lymphangiogenesis in the tumor-xenograft mouse models via controlling calreticulin expression. Boehme et al. have recently demonstrated that LECs exposed to prolonged pathologically elevated lymph flow exhibit hyperproliferative growth, which is mediated by elevated hypoxia-inducible factor (HIF)-1α expression and increased mitochondrial NOX-derived ROS production [98]. In addition, the authors report attenuated proliferation of LECs exposed to mitochondrial antioxidants, indicating the importance of mitochondrial ROS in driving LEC proliferation under mechanical stress. Under hypoxic conditions, tumor cells upregulate HIF expression and activity, which induce the transcription of various growth factors′ genes responsible for increasing angiogenesis and lymphangiogenesis [99]. All the above findings suggest that ROS regulates lymphatic permeability, contractility and drainage, and lymphangiogenesis. Further studies are required to investigate the role of ROS in specific diseases and cell types.

## 4. Role of Lymphatic Vessels in Various Pathologies

### 4.1. Tumor Metastasis

**Figure 2:**
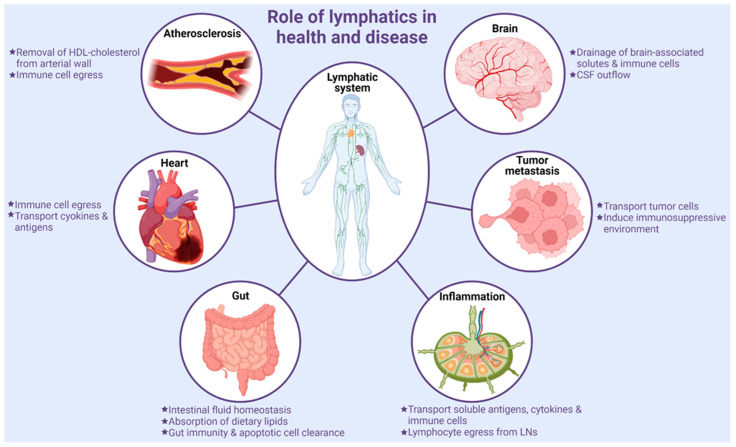
Role of the lymphatic system in health and disease.

Lymphatics serve as a route for cancer progression and metastasis. Secretion of well-known lymphangiogenic factor vascular endothelial growth factor (VEGF)-C and other growth factors by tumor cells lead to increased LV density in tumor tissues [100]. Tumor-associated lymphatics aid in the drainage of interstitial fluid containing various cell types and tumor-cell-originated macromolecules to sentinel LNs. Metastatic cells that detach from the primary tumor readily enter into highly permeable surrounding LVs and reach distant organs (Figure 2). Previous studies have indicated positive correlations between the levels of VEGF-C, VEGF-D, and VEGFR3 with an increased incidence of LN and distant organ metastasis [101,102,103]. In addition to metastasis, angiogenesis and lymphangiogenesis support tumor growth by other mechanisms. For example, the blood vascular system supplies nutrients and oxygen to the tumor hypoxic environment, while inflamed lymphatics induce an immunosuppressive microenvironment via reducing dendritic cell (DC)-mediated cytotoxic lymphocyte function by producing T cell inhibitory programmed-death ligand 1 (PD-L1), transforming growth factor-β (TGF-β), iNOS), and indoleamine 2,3-dioxygenase (IDO), thereby stimulating tumor progression [104]. Moreover, tumor tissue LV density has been shown to correlate with poor prognosis in cancer patients [105,106,107]. A previous study employing photodynamic therapy with benzoporphyrin-derivative verteporfin to destroy tumor-associated LVs showed prevention of mouse melanoma cell metastasis and subsequent tumor relapse. In addition, the combination of photodynamic and anti-lymphangiogenic therapies further reduced the invasion of tumor cells into LVs [108]. Conversely, drainage of tumor antigen-carrying migratory DCs to draining LNs is required for an antitumor immune response [109,110]. In addition, Fankhauser et al., in their study using the mouse melanoma model, reported enhanced T cell infiltration and potentiation of immunotherapy with experimental induction of tumor lymphangiogenesis [111]. Similar beneficial effects of augmented intra-tumoral LV density have been shown in colorectal cancer [112]. Therefore, despite the role of LVs in promoting tumor metastasis, LVs are required for the generation of optimum antitumor immune response to potentiate immune therapies. Future studies are required to further explore the role of LVs in specific cancer types.

### 4.2. Inflammation

Peripheral LVs serve as the principal route for the transportation of soluble antigens, cytokines, and immune cells from peripheral tissue to draining LNs and aid in the resolution of inflammation. LNs possess discrete compartments for T- and B lymphocytes. LECs present in afferent LVs via the expression of various adhesion molecules and chemokines control the entry and transport of immune cells through LVs and their positioning within LNs. LECs secrete chemokines CCL21 and CCL19, which guide and help in homing of CCR7^+^ activated DCs, macrophages, T- and B lymphocytes to lymphatics and LNs [113,114,115], while LECs with low expression of podoplanin and CCL21 but high CCR10 ligand CCL27 and Duffy antigen receptor for chemokines (DARC) levels direct the movement of CCR10^+^ T-lymphocytes into LVs [116]. Medullary LECs present in LNs expressing self-antigens and PD-L1 are involved in the depletion of alloreactive CD8^+^ T cells [117]. Cell adhesion molecules-common lymphatic endothelial and vascular receptor-1 and mannose receptor 1, have been shown to regulate leukocyte trafficking via LVs [118]. Interestingly, a study suggested reduced maturation of DCs via interaction of DC CD11b with intercellular adhesion molecule 1 receptor present in inflamed LECs [119].

Sphingosine 1-phosphate (S1P), synthesized intracellularly by phosphorylation of sphingosine, is a metabolic intermediate linking sphingolipids to glycerophospholipids [120]. The levels of S1P vary between blood (∼1 μM) and lymph (∼0.1 μM). LEC-dependent S1P gradient within LNs is required for egress of T- and B-lymphocytes from LNs into efferent lymph and thus is important for lymphocyte recirculation [121]. Further, S1P enhances the survival of naïve T cells and regulates the organization of LV junctions [122,123]. Lysophosphatidic acid (LPA), a bioactive lipid, has also been reported to support lymphocyte trafficking [124]. Altogether, these discoveries support the pivotal role of lymphatics in controlling immune response and inflammation.

### 4.3. Gut Homeostasis and Inflammatory Bowel Disease

Intestinal LVs include lacteals present in intestinal villi and mesenteric collecting LVs and constitute the largest lymphatic bed of the human body. These vessels aid in the drainage of lymph from gastrointestinal and lumbar regions into cisterna chyli and promote intestinal homeostasis [125]. Intestinal LVs also play crucial roles in the absorption of dietary lipids and the maintenance of gut immunity [126]. In adults, most of the lymphatics are quiescent under physiological conditions; however, lacteal LECs proliferate slowly and continuously, suggesting an ongoing lymphangiogenesis in adult lacteals [127]. A continuous lacteal regeneration may be a compensatory mechanism for consistent exposure to high lipoprotein levels, other biologically active dietary and microbial products, osmolarity gradients, and high mechanical strain originating from gut peristalsis and villus contractions [128,129,130]. Despite knowing the important role of intestinal lymphatics, the molecular factors regulating maintenance, renewal, and above functions of intestinal lymphatics are not fully understood.

Intestinal LVs are pivotal for gut immunosurveillance involved in inducing mucosal immunity and tolerance. These vessels regulate homing of CCR7^+^ DCs to mesenteric LNs in response to LEC-secreted CCL21, thereby establishing oral tolerance [131]. The intestinal epithelium has a high renewal rate and renews every 4–5 days in rodents and 5–7 days in humans. Intestinal LVs help in the transportation of apoptotic intestinal epithelial cells to mesenteric LNs and induce regulatory T (Treg) cells [132]. There are distinct mesenteric LNs to which different regions of the intestine drain and orchestrate adaptive immune responses specific to different intestinal segments [133].

Inflammatory bowel disease (IBD), including Crohn′s disease (CD) and ulcerative colitis (UC), is characterized by chronic intestinal inflammation. Its primary causes include genetic factors, dysregulation in immune response, diet, and other environmental cues [134]. Increased permeability of blood vessels in CD leads to accumulation of interstitial fluid that results in enhanced lymph flow to prevent edema. In human IBD patients, studies have reported augmented lymphangiogenesis, LV obstruction, dilation, and submucosal edema [135,136,137]. The presence of obstructive lymphoid aggregates consisting of T cells, B cells, and macrophages formed within lymphatic vasculature and expansion of mesenteric fat on the intestinal wall (also called creeping fat) are the major characteristics observed in patients with CD and result from impaired lymphatic drainage [138,139,140].

### 4.4. Lymphatics in Neurological Disorders

Under normal physiological conditions, the brain parenchyma is devoid of LVs, and clearance of cellular debris and waste products of the brain are mediated by the glymphatic system [141]. This system was proposed to aid in the exchange of small molecules between cerebrospinal fluid (CSF) and interstitial fluid [142,143]. Recent studies have characterized the architecture and importance of LVs present in the brain and spinal cord meninges [144,145,146,147]. In contrast to most peripheral lymphatic vasculature, which develops during mouse embryonic stage, meningeal LVs mature postnatally [148]. In adults, the development and maintenance of meningeal LVs depend on VEGF-C/VEGFR3 signaling [148]. The development, cytoarchitecture and morphology of meningeal LVs have been reviewed recently [149]. Meningeal LVs are identified as the major route of CSF outflow into deep and superficial cervical LNs in both humans and rodents [145,150,151,152,153]. These lymphatics are also required for the drainage of brain-associated interstitial fluid, solutes, and various immune cells (lymphocytes and CD11c^+^ CCR7^+^ DC) egress from CSF to the peripheral lymphatic system [145,151,153,154]. Citing the role of meningeal LVs in the above-mentioned processes, these lymphatics are involved in the pathogenesis of various age-related neurodegenerative diseases, such as Alzheimer′s disease. Ablation of meningeal LVs in mouse models of Alzheimer′s disease has been shown to increase brain β-amyloid levels and promotes its deposition in the meninges, demonstrating the role of these vessels in the clearance of β-amyloids [145,155]. Further, meningeal LVs are essential for the removal of extracellular tau aggregates from the central nervous system (CNS), which is another neuropathological hallmark of Alzheimer′s disease [156]. In addition, impaired meningeal lymphatic drainage leads to the accumulation of brain α-synuclein aggregates in Parkinson′s disease models [157,158] and induces neuroinflammation [159,160].

Contrary to the beneficial roles of meningeal LVs in Alzheimer′s and Parkinson′s disease and attenuating neuroinflammation, these lymphatics contribute to the pathogenesis of autoimmune demyelinating disease of the CNS, multiple sclerosis. Further, by serving as a route for the drainage of neurological immune cells and antigens to cervical LNs, meningeal LVs may support CNS infection [153]. All these studies demonstrate the key role of lymphatics in neurological pathologies.

### 4.5. Cardiovascular Disease

Atherosclerosis is a vascular inflammatory disease characterized by the accumulation of lipids in large and medium-sized arteries. Hoggan et al. first reported the presence of LVs in the arterial wall >100 years ago [161]. Subsequent studies described the existence of lymphatics in the blood vessels of dogs, pigs, and humans [162,163,164,165,166]. Despite the presence of LVs in the arterial wall, the functional role of arterial lymphatics in atherosclerosis development has not been investigated until recently. Martel et al. demonstrated that these arterial LVs play a critical role in the removal of high-density lipoprotein cholesterol from atherosclerotic arteries and skin via macrophage reverse cholesterol transport (mRCT) [167]. Lymphatic insufficiency has been shown to elevate plasma cholesterol levels [168]. In addition, hypercholesterolemia downregulates the expression of lymphangiogenic factors, including VEGF-C, Ang2, and FoxC2 in peripheral tissues, and impairs lymphatic drainage [169]. ApoA-I and VEGF-C152S treatments have been shown to improve lymphatic transport and attenuate atherosclerotic lesion formation [170,171]. In addition, a recent study by Singla et al. demonstrated the inhibition of atherosclerosis and reduced accumulation of fluorescently-labeled cholesterol in the left carotid artery with improved arterial drainage to periarterial LNs [21]. The role of lymphatics in cholesterol transport has been reviewed in the past [172]. Dissections of the left carotid artery draining deep cervical LNs and surrounding LVs in ApoE^-/-^ mice have been observed to augment atherosclerosis and promote the accumulation of CD3^+^ T cells in the intimal and adventitial arterial layers [173]. In addition, inhibition of lymphangiogenic VEGFR3-mediated signaling led to T cell enrichment in atherosclerotic lesions, suggesting the role of arterial lymphatics in the egression of arterial T cells. Taken together, these studies suggest that lymphatics play a beneficial role in arterial cholesterol efflux and inflammatory cell egress, which suppresses atherosclerosis development [174].

Blockade of coronary arteries due to atherosclerosis is responsible for the reduced supply of blood to the cardiac muscles, consequently leading to cardiac tissue damage, cell death, and heart failure. This process is accompanied by increased permeability of myocardial microvasculature and results in interstitial fluid accumulation (myocardial edema) and elevated lymph flow [175,176]. Previous studies have suggested that impairment of cardiac lymphatic drainage stimulates cardiac edema, and stimulated cardiac lymphangiogenesis associates with improved cardiac function via supporting the resolution of inflammation and reducing edema [177,178,179,180]. This information advocates that stimulated cardiac lymphangiogenesis may be a novel and potential therapeutic approach for treating cardiac disease.

### 4.6. Lymphedema

Tissue edema caused by an accumulation of protein-rich fluid due to impaired lymphatic drainage is referred to as lymphedema. It mostly occurs in limbs but can also affect the chest wall, abdomen, neck, and genital regions. Primary lymphedema is a genetic disease, e.g., Nonne–Milroy disease and Meige disease [181]. Secondary lymphedema is usually caused by infection (*Wuchereria bancrofti*) and surgical and radiotherapy procedures [182,183,184]. These diseases result from insufficient lymph drainage.

## 5. Conclusions

LVs have significantly different structures and functions from blood vessels due to their roles in the maintenance of tissue fluid homeostasis, uptake of dietary lipids and macromolecules, and immunosurveillance. Significant advances have been made in the last decade regarding the molecular mechanisms regulating LV formation. Low levels of ROS are required for lymphangiogenesis, while excess generation of ROS contributes to inhibition of lymphangiogenesis and impaired lymphatic drainage. However, the effects of ROS derived from LECs and other neighboring cell types (s) in regulating lymphangiogenesis are understudied. Existing data support a pivotal role of NOX-derived ROS in regulating LV formation, and future mechanistic studies are necessary to identify novel molecular stimulators of NOXs, investigate the mechanisms of NOX activation and downstream targets of ROS, and examine the effects of antioxidants in modulating lymphangiogenesis. Understanding these mechanisms may provide new potential therapeutic targets for the treatment of lymphatic-related pathological disorders.

## Figures and Tables

**Table 1:** Types and generation of reactive oxygen species.

Name of Molecule	Half-Life of Molecule	Generation of Molecule
Nitric oxide (NO^•^)	10^−5^ to 10^−3^ s	*Nitric oxide synthase* L-arginine + O_2_ + NADPH → L-citrulline + NO^•^ + NADP^+^ *Reduction of nitrite* *Deoxyhaemoglobin/myoglobin* NO_2_^−^ + Fe^2+^ + H^+^ → NO^•^ + Fe^3+^ + OH^−^ *Xanthine oxidoreductase* NO_2_^−^ + Mo^4+^ + H^+^ → NO^•^ + Mo^5+^ + OH^−^ *Protons* NO_2_^−^ + H^+^ → HNO_2_ 2 HNO_2_ → 2 N_2_O_3_ + H_2_O N_2_O_3_ → NO^•^ + •NO_2_ *Ascorbate* NO_2_^−^ + H^+^ → HNO_2_ 2 HNO_2_ + Asc → 2 NO^•^ + dehydroAsc + 2 H_2_O *Polyphenols (Ph-OH)* NO_2_^−^ + H^+^ → HNO_2_ Ph-OH + HNO_2_ → Ph-^•^O + NO^•^ + H_2_O
Superoxide (O_2_^•−^)	10^−11^ to 10^−9^ s	*NADPH oxidase* NADPH + 2O_2_ → NADP^+^ + 2O_2_^•−^ + 2H^−^ *Xanthine oxidase* Hypoxanthine + H_2_O + 2O_2_ → Xanthine + 2O_2_^•−^ + 2H^−^ Xanthine + H_2_O + 2O_2_ → Uric acid + 2O_2_^•−^ + 2H^−^ *Uncoupled endothelial nitric oxide synthase* NADPH + 2O_2_ → NADP^+^ + 2O_2_^•−^ + 2H^−^ *Mitochondrial electron transport chain complexes I and III* O_2_ → O_2_^•−^ *Lipooxygenase* Arachidonic acid + O_2_ → HPETE+ O_2_^•−^
Hydroxyl radical (^•^OH)	10^−9^ s	*Fenton reaction* Fe^2+^ + H_2_O_2_ → Fe^3+^ + ^•^OH + OH^−^ *Haber-Weiss reaction* ^•^O_2_^−^ + H_2_O_2_ → ^•^OH + OH^−^ + O_2_ HOONO → ^•^OH + NO_2_^•^
Lipid peroxyl radical (LOO^•^)	7 s	L-H + ^•^X → L^•^+ XH LOO^•^ + L-H → LOOH + L^•^ L^•^ + O_2_ → LOO^•^ L-H: polyunsaturated fatty acid ^•^X: oxidizing character (i.e., ^•^OH or O_2_^•−^) L^•^: lipid radical
Peroxynitrite (ONOO^−^)	10^−2^ s	O_2_^•−^ + NO^•^ → ONOO^−^
Hydrogen peroxide (H_2_O_2_)	10^−8^ (in presence of catalase) or 10^−3^ s	2O_2_^•−^ + 2H^+^ → H_2_O_2_ + O_2_
Hypochlorous acid (HOCl)	<1 min	*Myeloperoxidase* H_2_O_2_ + Cl^−^ → HOCl + OH^−^
Ozone (O_3_)	1 min	x^1^O_2_ + yH_2_O 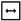 [H_2_O_3_(y−1)H_2_O] → H_2_O_2_ + (x−1)^3^O_2_ + O_3_

## Data Availability

Not applicable.
